# Effects of Head-Only Exposure to 900 MHz GSM Electromagnetic Fields in Rats: Changes in Neuronal Activity as Revealed by c-Fos Imaging without Concomitant Cognitive Impairments

**DOI:** 10.3390/biomedicines12091954

**Published:** 2024-08-27

**Authors:** Bruno Bontempi, Philippe Lévêque, Diane Dubreuil, Thérèse M. Jay, Jean-Marc Edeline

**Affiliations:** 1 Institut de Neurosciences Cognitives et Intégratives d’Aquitaine, CNRS UMR 5287, Université de Bordeaux et Ecole Pratique des Hautes Etudes, 33000 Bordeaux, France; bruno.bontempi@u-bordeaux.fr; 2 XLIM, CNRS UMR 6172, Université de Limoges, 87060 Limoges, France; philippe.leveque@unilim.fr; 3 Institut des Neurosciences Paris-Saclay (NeuroPSI), Université Paris-Saclay, CNRS, CEA Paris-Saclay, bât 151, 91400 Saclay, France; diane.dubreuil@gmail.com; 4 Institut de Psychiatrie et Neurosciences de Paris, UMR_S 1266 INSERM, Université Paris Cité, 75014 Paris, France; therese.jay@inserm.fr

**Keywords:** spatial learning, immunohistochemistry, rat, radio frequency

## Abstract

Over the last two decades, animal models have been used to evaluate the physiological and cognitive effects of mobile phone exposure. Here, we used a head-only exposure system in rats to determine whether exposure to 900 MHz GSM electromagnetic fields (EMFs) induces regional changes in neuronal activation as revealed by c-Fos imaging. In a first study, rats were exposed for 2 h to brain average specific absorption rates (BASARs) ranging from 0.5 to 6 W/kg. Changes in neuronal activation were found to be dose-dependent, with significant increases in c-Fos expression occurring at BASAR of 1 W/kg in prelimbic, infralimbic, frontal, and cingulate cortices. In a second study, rats were submitted to either a spatial working memory (WM) task in a radial maze or a spatial reference memory (RM) task in an open field arena. Exposures (45 min) were conducted before each daily training session (BASARs of 1 and 3.5 W/kg). Control groups included sham-exposed and control cage animals. In both tasks, behavioral performance evolved similarly in the four groups over testing days. However, c-Fos staining was significantly reduced in cortical areas (prelimbic, infralimbic, frontal, cingulate, and visual cortices) and in the hippocampus of animals engaged in the WM task (BASARs of 1 and 3.5 W/kg). In the RM task, EMF exposure-induced decreases were limited to temporal and visual cortices (BASAR of 1 W/kg). These results demonstrate that both acute and subchronic exposures to 900 MHz EMFs can produce region-specific changes in brain activity patterns, which are, however, insufficient to induce detectable cognitive deficits in the behavioral paradigms used here.

## 1. Introduction

Over the last two decades, the rapid expansion of mobile communication has raised concerns about the possible deleterious health effects of electromagnetic fields (EMFs) generated both by relay stations and cellular phones [1,2,3]. Although the biological effects of EMFs remain controversial, the brain is likely to be a primary target of EMFs because cellular phones are usually held close to the head while in use. A large diversity of effects has been reported following exposure to various energy levels and types of EMFs which, sometimes, are quite different from those used in global systems for mobile (GSM) communication. In humans, exposure to either low- or high-frequency EMFs has been suspected to increase the risks of cancer [4,5,6] and of leukemia [7,8]. Impairments of cognitive functions [9,10], changes in electroencephalogram [11,12,13,14,15,16], and a higher occurrence of headaches [17,18] have also been reported. In animals, where EMF exposure conditions can be more rigorously controlled, some biological effects have also been documented. Changes in electrophysiological activity [19], in neurotransmitter content [20,21,22,23,24], and in glial reactivity [25,26,27] have been described. However, other studies did not report significant effects: brief or prolonged exposures to EMFs were shown to have a negligible effect on the genomic response of brain cells [28,29]. Effects on blood–brain barrier permeability are more controversial: some studies found no effects [30,31], whereas other studies found modifications of permeability [32,33,34,35]. 

A lack of consensus is also striking in the field of cognitive neuroscience: Some studies have shown an impairment of learning and memory processes in humans [10] and animals [36,37,38], whereas others failed in reproducing significant deleterious effects [39,40,41,42,43,44,45,46,47] or even observed beneficial effects of EMF exposures either in normal [48] or in pathological conditions [49,50]. Several factors could potentially explain these discrepancies, in particular the EMF frequencies and intensities, but also the exposure conditions. Indeed, testing rats with the same behavioral tasks, some studies have used whole-body exposures [36,37,38], whereas others have selected head-only exposures (e.g., see [42,43]), with this latter situation better mimicking the exposure to cellular phones in humans.

The aim of this study was to determine if acute or subchronic exposure to GSM EMFs could induce detectable changes in neuronal genomic response by measuring changes in the expression of the immediate early gene c-Fos. This gene is widely used as an indirect marker of neuronal activity and is also involved in the control of target genes playing a crucial role in the long-term reorganization of brain circuits during various pathogenic and non-pathogenic situations. Using c-Fos imaging, our goal was to identify the different brain regions which may be the most sensitive to the effects of EMF exposures. 

In a first experiment, we describe the consequences of a single exposure, performed during resting conditions, for groups of rats submitted to brain averaged specific absorption rate (BASAR) ranging from 0.5 to 6 W/kg (i.e., from below the international limit of 2 W/kg up to three times this value). In a second experiment, we investigated the consequence of a subchronic exposure (14 days), performed before behavioral tests, using groups of rats submitted to two BASARs (1 and 3.5 W/kg). The rationale was to evaluate if repeated exposures may lead either to larger effects than a single exposure (by cumulating effects occurring after single exposure) or to smaller effects than a single exposure (by adaptation to EMF exposure). In addition, engaging the animals in a learning task made it possible to pinpoint both increases and decreases in c-Fos labelling whereas only increases are usually observable in resting conditions due to the very low constitutive expression of c-Fos (floor effect). 

## 2. Materials and Methods

### 2.1. Animals

Naïve male Sprague Dawley rats (150–180 g at their arrival in the laboratory, Charles River, L’Arbresle, France) were housed in a humidity (50–55%) and temperature (22–24 °C)-controlled facility on a 12 h/12 h light/dark cycle (lights on at 6:30 a.m.) with free access to food and water, except during behavioral testing. Animals were group-housed (two to five per cage) and were allowed a 1-week period of habituation to the colony room before testing. They were handled and weighed every day. At the end of the habituation period, rats were single housed. The analysis of the effects of acute EMF exposure (Experiment 1) was conducted in animals that had ad libitum access to food and water. All the groups of animals used for memory testing (Experiment 2) were progressively food-restricted and maintained to 85% of their free-feeding weight. All procedures were performed in conformity with French (JO 887–848) and European (86/609/EEC) legislations on animal experimentation.

### 2.2. Electromagnetic Field Exposure System

**Figure 1:**
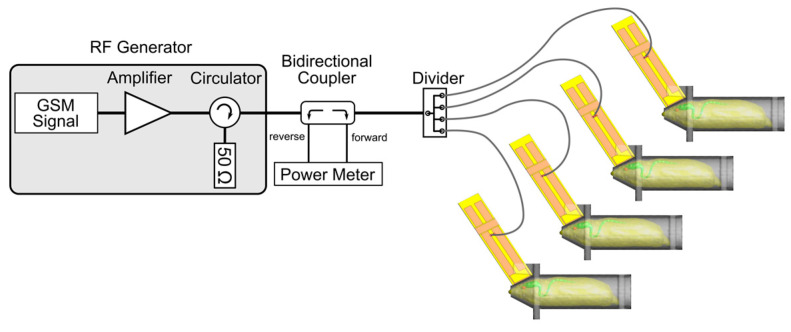
The exposure system setup included a 900 MHz radiofrequency generator emitting a GSM EMF pulsed at 217 Hz, a power meter, and a 4-output power divider connected to individual loop antennas enabling a head-only exposure of 4 animals simultaneously.

The exposure system was the one used in previous experiments [42,43] (see for details [51]). Briefly, a radiofrequency generator (RFPA S.A., model RFS9001800-25), emitting a GSM EMF (1/8 duty factor) pulsed at 217 Hz (pulse emission during 576 sec every 4.6 ms) was connected to a 4-output divider, allowing exposure of four animals simultaneously. Each output was connected to a loop antenna enabling a local exposure of the animal’s head (Figure 1). The loop antenna consisted of a printed circuit, with two metallic lines engraved in a dielectric epoxy resin substrate (dielectric constant εr = 4.6). At one end, this device consisted of a 1 mm width line forming a loop placed close to the animal’s head. 

Brain average specific absorption rates (BASARs) were determined numerically using a numerical rat model with the Finite Difference Time Domain method and experimentally in homogeneous rat phantom using a Vitek or Luxtron probe for the measurement of temperature rises. In this case, BASARs, expressed in W/kg, were calculated using the following relation: SAR = C∆T/∆t, with C being the calorific capacity in J/(kg∙K), ∆T the temperature variation in K, and ∆t the time variation in seconds. Comparisons were made between numerical and experimental specific absorption rate (SAR) values in homogeneous phantoms, especially in the equivalent rat brain area. The agreement among numerical and experimental SAR values was good (see for details [51]). Finally, the BASAR in a non-homogeneous rat model was obtained: 6.8 W/kg for one Watt incident power. The incident power was than adjusted to obtain the experimental BASAR levels used in our experiments.

EMF exposure and behavioral testing were performed in two different rooms. During radiofrequency exposure, rats were restrained in rockets made of 5 mm thick transparent Plexiglas and consisting of a cylinder (6 cm in diameter, 15 cm long) and a truncated cone (3 cm long) in which the rat’s head was inserted (see for details [42,43,51]). The loop antenna was placed against the cone directly above the rat’s head. The end of the cone was opened, and holes were made in the rocket’s cylinder to facilitate breathing and minimize sweating and increases in the animal’s body temperature. A Plexiglas disk was secured at the entry of the cylinder to prevent the rat from backing out of the rocket and to minimize as much as possible any movement of the head. To prevent interference from one antenna to another, rockets were separated from each other by a double layer of absorbing material (APM 12, Hydral, 12 cm thick).

### 2.3. Habituation to Restrained Conditions

To minimize the nonspecific effects of stress induced by the rocket confinement on learning performance as well as on c-Fos expression, animals were first submitted to a habituation protocol. Rats were placed in the rockets with the loop antenna disconnected from the radiofrequency generator. They were adapted gradually to the restrained conditions for either 14 days (Experiment 1) or 7 days (Experiment 2). Also, on the first day after arrival in the laboratory, rockets were introduced in the home cages, allowing animals to enter freely in them. During the following days, confinement in the rockets was progressively increased from 5 min to 2 h (Experiment 1) or from 5 min to 45 min (Experiment 2). Across the days, the animal movements in the rockets were less frequent, attesting that the restrained conditions were relatively well tolerated. In a few cases (n = 3), animals showing important movements in the rocket at the end of this habituation period (they moved for more than 25% of the time spent in the rocket) were discarded from the study.

### 2.4. Experiment 1

This experiment was designed to investigate the effects of 900 MHz EMF exposure on the basal level of neuronal activity. Awake, “resting” animals (maintained in their home cage prior to being placed in the rockets) were used. Following the 2-week habituation to the rockets, animals were randomly assigned to 6 different treatment groups (n = 9/group): one sham-exposed group immobilized for 2 h in the rockets but without EMF and five experimental groups exposed for 2 h to 900 MHz EMF at BASARs of 0.5, 1, 2, 4, and 6 W/kg. This range of BASARs was selected because it started below the international limit up to 3 times this value (the SAR limit is set by international requirements and by the Council of the European Union at 2 watts/kilogram averaged over 10 grams of body tissue). The exposure duration was set at 2 h, which is probably the longest time a rat can accept restrained conditions in the rocket used for the exposure. It is also close to the average time per day for extensive cell phone users.

Immediately at the end of the exposure period, the animals were deeply anesthetized and perfused, and their brains were processed for immunocytochemistry as described below.

### 2.5. Experiment 2

#### 2.5.1. Aim and Groups

This experiment was designed to determine the effects of EMF exposures on c-Fos expression while rats were submitted to learning tasks. Animals were engaged in behavioral tasks to allow for (i) detection of potential decrease in neuronal activity that would be difficult to observe in basal state and (ii) possible changes in the activation of brain areas in response to cognitive demand. Animals were randomly assigned to four different treatment groups (n = 12/group). Cage control rats were maintained in their home cage and did not receive any treatment before being submitted to the learning task. Sham-exposed rats were immobilized for 45 min in the rockets immediately prior to each daily training session but without EMF exposure. Two experimental groups were exposed to 900 MHz EMF at BASAR of either 1 or 3.5 W/kg for 45 min immediately prior to each daily training session. The BASAR of 1 W/kg was selected based on the results of Experiment 1, and the BASAR of 3.5 W/kg was the highest value our system could deliver when 4 animals were simultaneously exposed. There was a time-lag of 20 min between the beginning of exposure of the four animals (i.e., they were placed in the rockets every 20 min), thus allowing to test each rat immediately at the end of the EMF exposure or the sham exposure. Time of testing in the day (morning or afternoon) was counterbalanced between groups. The equipment and the behavioral procedures used in the two memory tasks were exactly the same as those described in our previous experiments [43,52]. They are only briefly summarized here. Based upon our previous studies [42,43,52], we determined that the asymptotic level of performance was reached after 10 days in the working memory task and after 14 days in the reference memory task. Accordingly, we chose an exposure period of 10 days and 14 days for the working memory task and the reference memory task, respectively. Note also that because the animals were restrained in Plexiglas rockets designed to accommodate rats of a maximum weight of 250 g, we could not extend the duration of the experiments.

#### 2.5.2. Spatial Working Memory Task

This task was conducted in a radial maze made of an octagonal central platform (23 cm in diameter) from which radiated eight arms (80 cm long, 12 cm wide) in a symmetrical fashion. At the end of each arm, a food cup (5 cm in diameter, 1 cm in height) could be baited with food rewards (i.e., chocolate cereals). The room was illuminated by a neon light located above the apparatus, thus avoiding shadows within the maze. Large cues were placed on the walls of the room and served as spatial distal cues. In order to be familiarized with the radial maze and its environment, rats were first submitted to habituation sessions immediately after the last 3 days of adaptation to restrained conditions. After the restraint conditions in the rockets, a group of six rats was placed for 20 min in the maze where food rewards were scattered throughout the maze to encourage exploration. On the last day of habituation, rats explored the maze in groups of three, and food rewards were placed only in the food cup of each arm. This habituation session was terminated when all eight arms were visited, and all food rewards were consumed. Spatial working memory testing sessions began the next day and lasted for 10 consecutive days. Rats were submitted to a daily session, during which all arms were baited. Each session consisted of one trial and started by placing the rat in an opaque Plexiglas cylinder located in the center of the maze platform. After 10 s, the cylinder was removed, and the rat was allowed to freely explore the maze until all eight arms had been visited or when the rats had made a total of 16 visits or when 10 min had elapsed. Arms were not rebaited during the ongoing trial, so repeated entries into an arm were counted as working memory errors. Choice accuracy was also evaluated by the rank of the first error, which was defined as the number of consecutive correct visits in a training session before the first error occurred (i.e., the maximum rank was 8). Rats learning this task typically show a progressive decrease in the number of working memory errors over days while the rank of the first error increases. Seventy to ninety minutes after the end of the 10th session, half of the animals randomly selected (n = 6/groups) were deeply anesthetized and perfused, and the brains were processed for immunocytochemistry (see below). The selection of these animals was done blind to their behavioral performance.

#### 2.5.3. Spatial Reference Memory Task

Reference memory performance was measured using a dry-land version of the Morris water maze [43,53,54]. This spatial navigation task was conducted using a circular arena (120 cm in diameter) surrounded by a transparent plastic edge (10 cm high). The arena was mounted on a rotating pedestal, 70 cm above the floor. Thirteen white opaque circular boxes (3 cm in diameter), which were glued on the arena, defined a regular geometrical pattern in the arena. Each box was covered by a white lid. Chocolate cereals used as food rewards were placed in selected boxes. During the last 4 days of adaptation to restrained conditions in the rockets, rats were familiarized with the boxes placed in the arena. On the first day, rats were habituated to eat the food reward contained in a box placed in their home cage. On the two following days, the box was covered with the lid, and the rats had to learn to open it to get the food reward. Rats were given four trials/day for two consecutive days. On the second day, rats had to open the box in less than 10 s, otherwise four additional trials were given. On the fourth habituation day, rats were placed by group of three on the circular platform and were free to explore the non-baited apparatus for 15 min. The spatial reference memory testing session begun the next day and lasted for 14 consecutive days. Each rat had to learn that the food reward was located in only one box. For a given animal, the baited box was always located at the same position with regard to the reference space formed by the geometrical cues scattered around the room. Rats were submitted to a daily session made of four consecutive trials separated by a 1 min intertrial interval. The rat was placed on the platform at each of four different starting positions (North, South, East, and West) facing the transparent wall of the platform. From day to day, the order of the starting positions was randomly changed to prevent the rats from learning a specific route. To prevent strategies based on olfactory cues, between each trail and each rat, the platform was randomly turned by 60, 120, 180, 240, and 300 degrees. Each trial lasted a maximum of 5 min. If the rat did not find the goal box after 5 min, it was gently guided in front of the goal box and allowed to open it and eat the food reinforcement. Reference memory errors were defined as the number of boxes, other than the goal box, that the rat opened during a trial. A hit was achieved when the rat opened the goal box directly without opening any other box; on a given day, the maximum number of hits was four. Latency to open the goal box was also measured. Seventy to ninety minutes after the end of the 14th session, half of the animals randomly selected (n = 6/groups) were deeply anesthetized and perfused, and the brains were processed for immunocytochemistry (see below). The selection of these animals was done blind to their behavioral performance.

### 2.6. c-Fos Immunocytochemistry

Following deep anesthesia, rats were perfused transcardially with 350 mL of 0.9% NaCl followed by 350 mL of cold 4% paraformaldehyde in 0.1 M phosphate buffer (PB), pH 7.4. Brains were dissected, post-fixed for 12 h in the same fixative and cryoprotected in 30% sucrose/PB, and left overnight at 4 °C. They were then frozen, and 50 µm frontal sections were cut on a freezing microtome. Immunohistochemistry was performed on free-floating sections using a standard avidin–biotin (ABC Standard Elite kit, Vector Laboratories, Burlingame, CA, USA) method. The primary antibody was a rabbit polyclonal antibody (Ab-5, Oncogene Science, Cambridge, MA, USA, 1:20,000) raised to a synthetic peptide derived from aminoacid sequences 4-17 of the Fos protein. Incubation in 2% goat serum containing 0.2% Triton X-100 (Sigma, Setagaya City, Tokyo) and the primary antibody occurred overnight at room temperature on a rotating shaker. Following a series of 4 washes in PB, sections were incubated for 2 h at room temperature in a solution containing a biotinylated goat anti-rabbit secondary antibody (Jackson Immunoresearch, West Grove, PA, USA, 1:2000). Sections were washed again and processed with the avidin-biotin complex for 2 h at room temperature. Diaminobenzidine (0.05% solution, Sigma) was used as a chromogen and was revealed using a few drops of fresh 0.3% hydrogen peroxide solution. The reaction was stopped by washing in PB. Sections were mounted on gelatin-coated slides, dehydrated through a series of graded alcohols baths, and coverslipped.

### 2.7. Image Analysis and Cell Counting

**Figure 2:**
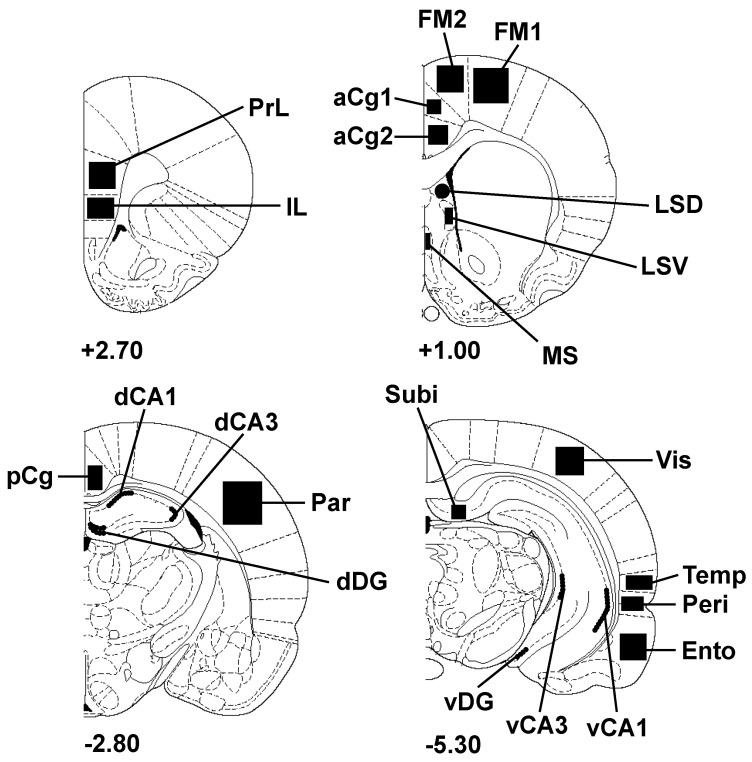
Schematic drawings of rat brain coronal sections showing the regions of interest (filled areas) selected for c-Fos imaging (adapted from the atlas of Paxinos and Watson [55]). Numbers indicate the distance in millimeters of the sections from bregma. aCg1: anterior cingulate cortex, area 1; aCg2: anterior cingulate cortex, area 2; dCA1: dorsal hippocampus, CA1 field; dCA3: dorsal hippocampus, CA3 field; dDG: dorsal dentate gyrus; Ento: entorhinal cortex; FM1: frontal cortex, primary motor area 1; FM2: frontal cortex, secondary motor area 2; IL: infralimbic cortex; LSD: lateral septum, dorsal part; LSV: lateral septum, ventral part; MS: medial septum; Par: parietal cortex; pCg: posterior cingulate cortex; Peri: perirhinal cortex; PrL, prelimbic cortex; Subi: subiculum; Temp: temporal associative cortex; vCA1: ventral hippocampus, CA1 field; vCA3: ventral hippocampus, CA3 field; vDG: ventral dentate gyrus; Vis: visual cortex, secondary lateral area.

Quantitative analysis of c-Fos-positive nuclei was performed using a color video camera (Sony^®^ DXC-950P, Sony, Tokyo, Japan) interfaced with an Olympus^®^ BX 50 microscope (Olympus, Tokyo, Japan). Fos-positive nuclei were counted in selected brain regions using computer-assisted software (Biocom Visiol@b^®^ 2000, version V4.50; ×20 magnification). Sampled areas defined according to the atlas of Paxinos and Watson [55] are presented in Figure 2. These sampled zones, which included most of the cortical areas (frontal, temporal, visual, parietal cortices) and several parts of the hippocampus, are areas receiving the highest SAR values (because of their proximity with the loop antenna). The number of c-Fos-positive nuclei measured in each region was counted bilaterally using a minimum of three consecutive sections in each brain by experimenters who were blind to the exposure conditions. The mean numbers of c-Fos-positive nuclei contributed by each animal in a group were averaged to generate the final means. Data are expressed as the number of c-Fos-positive nuclei/mm^2^ for all regions studied.

### 2.8. Statistical Analyses

Behavioral parameters and numbers of c-Fos-positive nuclei were compared using an analysis of variance (ANOVA) with repeated measures and, when appropriate, were followed by post hoc paired comparisons using the Newman-Keuls test. All data are presented as mean ± SEM. For all comparisons, values of *p* < 0.05 were considered as statistically significant.

## 3. Results

### 3.1. Experiment 1: Effect of EMF Exposure on Neuronal Activity as Revealed by c-Fos Imaging

**Figure 3:**
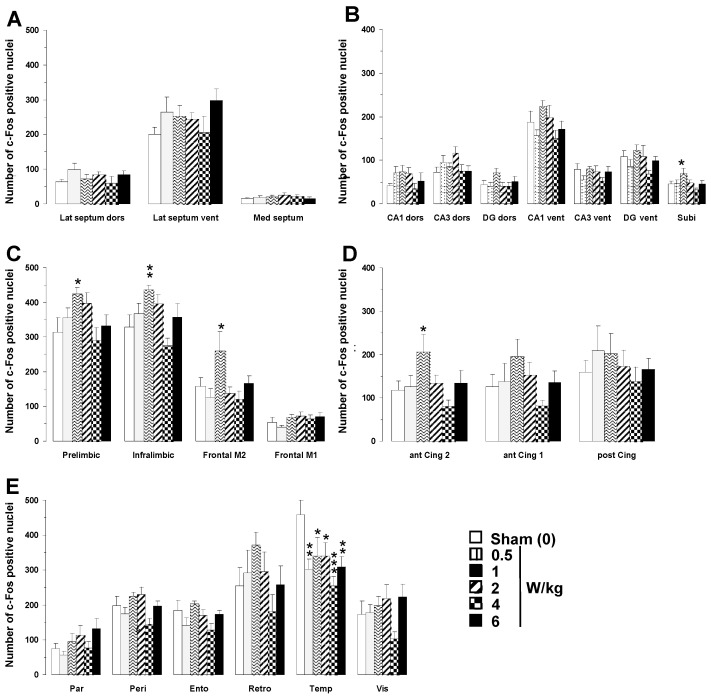
Effects of acute GSM 900 MHz electromagnetic field exposure on neuronal activity as measured in 23 brain areas by c-Fos immunohistochemistry in exposed and sham-exposed animals. Data are presented as number of c-Fos-positive nuclei in selected brain regions (mean ± SEM, n = 9/group). (**A**) Septal region; (**B**) hippocampal formation; (**C**–**E**) cortical regions. See Figure 2 for the list of abbreviations. Experimental animals were exposed for 2 h, while sham-exposed animals were placed in rockets but without any EMF exposure. * *p* < 0.05, ** *p* < 0.01, *** *p* < 0.001 for exposed versus sham-exposed animals; ANOVA followed by Fisher’s post hoc test.

A two-way ANOVA performed on c-Fos mapping data with BASARs as the between group factor and the 23 brain regions analyzed as the within-subjects factor indicated a significant group x region interaction (F(5, 1190) = 7.11; *p* < 0.0001), showing that region-specific changes in c-Fos expression occurred following EMF exposure (Figure 3). Analysis of individual regions revealed that EMF exposure induced a dose-dependent increase in c-Fos expression in the following brain regions: subiculum (F(5, 46) = 2.47; *p* < 0.05), prefrontal (prelimbic area, F(5, 46) = 2.49; *p* < 0.05; infralimbic area, F(5, 46) = 3.78; *p* < 0.01), frontal (M2 motor area, F(5, 46) = 2.73; *p* < 0.05), and anterior cingulate cortices (area 2, F(5, 46) = 2.51; *p* < 0.05). 

**Figure 4:**
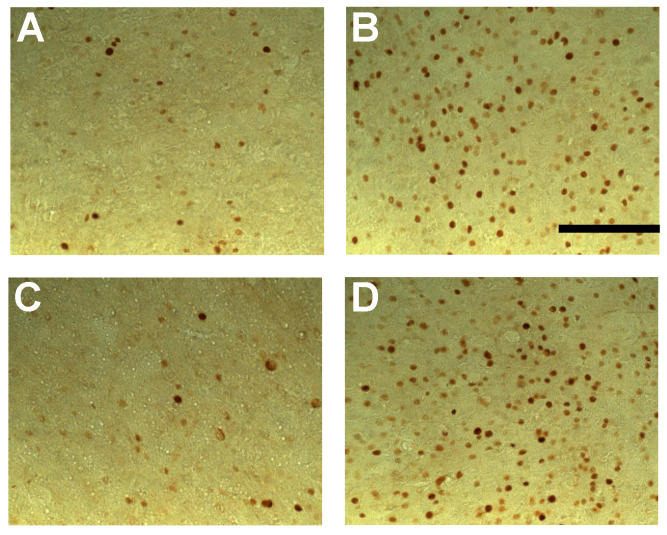
Photomicrographs (×20 magnification) of c-Fos immunoreactivity in coronal sections taken through the prelimbic cortex (**A**,**B**) and frontal cortex (area FM2; (**C**,**D**)) following acute GSM 900 MHz electromagnetic field exposure (SAR of 1 W/kg for 2 h). Note that this example represents the largest increased number of c-Fos-positive nuclei in exposed animals (1 W/kg; **B**,**D**) as compared to sham-exposed animals (0 W/kg; **A**,**C**). Scale bar: 100 µm.

Post hoc analyses revealed that only the BASAR of 1 W/kg significantly increased c-Fos expression as compared to sham animals in the subiculum (+53.2%, *p* < 0.05), prefrontal (prelimbic area +35.2%, *p* < 0.05; infralimbic area +32.9%, *p* < 0.01), frontal (M2 motor area +64.9%, *p* < 0.05), and anterior cingulate cortices (area 2, +74.2%, *p* < 0.05; Figure 2). A reduction in c-Fos expression was found in the temporal cortex at all BASARs (F(5, 46) = 3.26; *p* < 0.05), with the greatest decrease observed at the BASAR of 4 W/kg (−44.4%, *p* < 0.001; Figure 2). The perirhinal cortex was similarly affected (F(5, 46) = 2.87; *p* < 0.05) with a significant decrease observed at the BASAR of 4 W/kg (−27.3%, *p* < 0.05). Representative examples of the largest EMF-induced increase in c-Fos expression in the prefrontal and frontal cortices are presented in Figure 4. 

### 3.2. Experiment 2: Effect of EMF Exposure on Learning and Memory Performance and Associated Neuronal Activity

#### 3.2.1. Behavioral Data

In the following paragraphs, we present the behavioral results obtained from the subset of animals (n = 6 per group) used for the quantification of c-Fos labeling. In all but one cases, these results are similar to those obtained with the whole population of animals ran in the behavioral tests (n = 12 per group, see Supplementary Figures S1 and S2).

##### Working Memory Task

**Figure 5:**
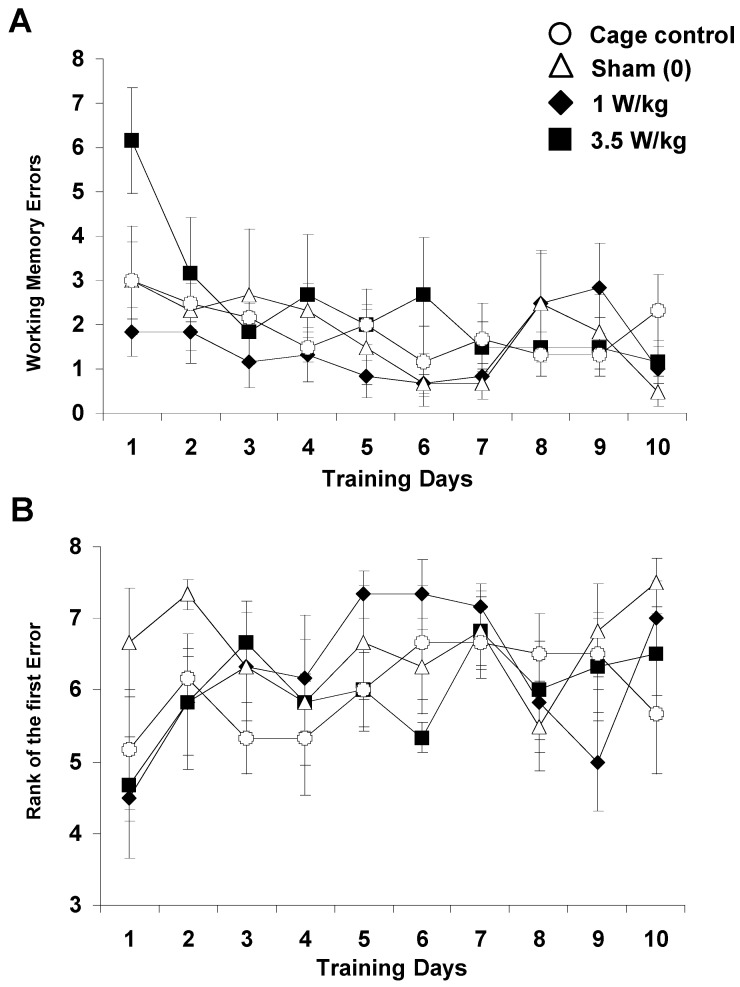
Effect of subchronic GSM 900 MHz electromagnetic field exposure on working memory performance as measured in the 8-arm radial maze. Data are presented as number of working memory errors (**A**) and rank of the first error (**B**) (mean ± SEM, n = 6/group) measured over 10 days of training. Cage control rats were not submitted to any treatment before each daily training session. Sham-exposed rats were placed in the rockets for 45 min without any EMF exposure. Experimental animals were exposed to EMFs for 45 min at SARs of either 1 or 3.5 W/kg. Note that for the rank of the 1st error, a high value indicates a better performance. No significant between-group differences were observed.

As shown in Figure 5A, EMF exposure did not significantly affect the number of working memory errors recorded over 10 days of testing (Treatment F(3, 20) = 2.06, *p* > 0.13, NS). For all groups, the number of working memory errors decreased significantly over the days (F(9, 180) = 3.08, *p* < 0.002), and the slope of the learning curve was similar across groups (Treatment x Day interaction: F(27, 180) = 1.06, *p* > 0.38, NS). Based on one-way ANOVA, each group, except the cage control group, improved its performance (*p* < 0.05). As illustrated in Figure 5B, the rank of the first error, which can be taken as a relevant index of working memory performance, was similar across groups (F(3, 20) = 0.74, *p* > 0.54, NS) and increased in a similar way over the days (Day: (F9, 180) = 2.54; *p* < 0.01); Treatment x Day interaction: F(27, 180) = 1.22; *p* > 0.22, NS). For all groups, one-way ANOVA indicated that the rank of first error increased across the days (*p* < 0.05). Latency to complete the task was also similar across groups (data not shown). The same results were obtained when analyses were performed on the entire population of animals used in this study (n = 12 per group, see Supplementary Figure S1). Overall, there was no evidence of an effect of EMF exposure in this task.

##### Reference Memory Task

**Figure 6:**
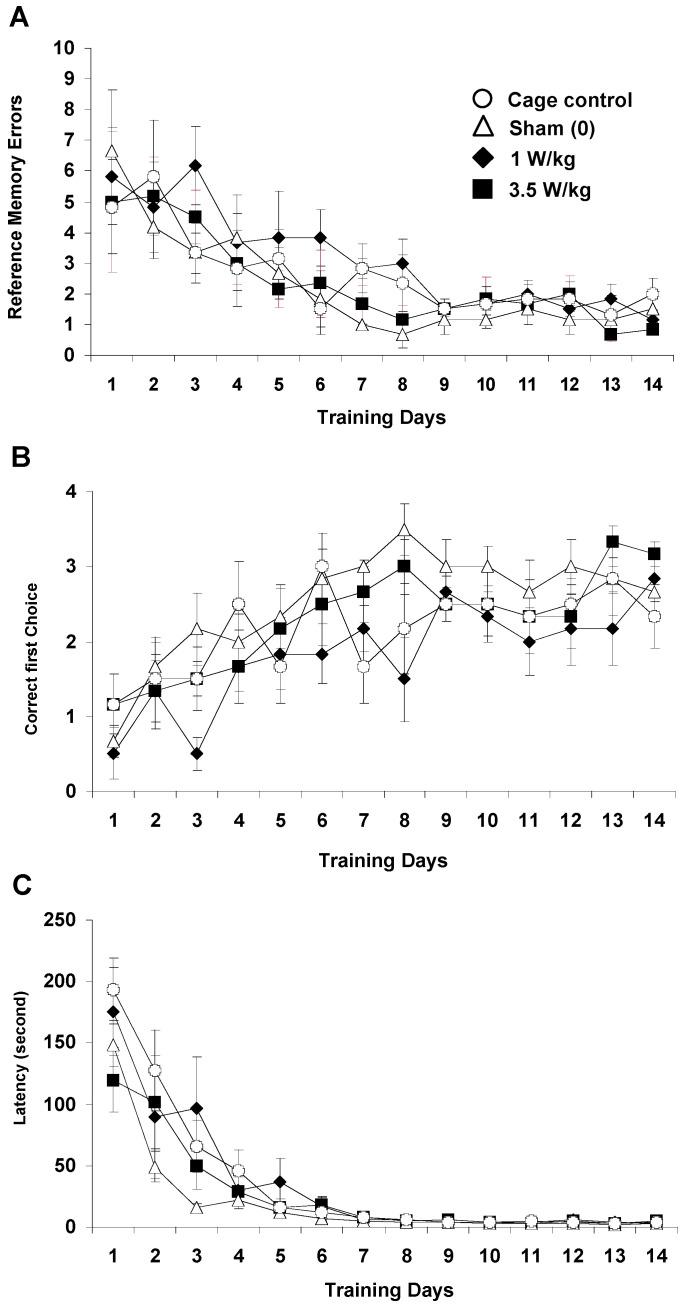
Effect of subchronic GSM 900 MHz electromagnetic field exposure on reference memory performance as measured in the spatial navigation task (dry-land version of the Morris water maze previously used in [43,53,54]). Data are presented as number of reference memory errors (**A**), number of hits (**B**), and latency in sec to find the baited box (**C**) (mean ± SEM, n = 6/group) measured over 14 days of training. Cage control rats were not submitted to any treatment before each daily training session. Sham-exposed rats were placed in the rockets for 45 min without any EMF exposure immediately prior to each testing session. Experimental animals were exposed to EMFs for 45 min at SARs of either 1 or 3.5 W/kg immediately prior to each testing session. No difference was observed in terms of number of errors (**A**) and latency to reach the goal box (**C**). In terms of number of hits (**B**), the group exposed to 1 W/kg differed from the sham-exposed group but did not differ from the cage control group. This effect on the number of hits was not found when larger sets of animals (n = 12 per group) were tested (see Supplementary Figure S2).

As shown in Figure 6A, EMF exposure did not significantly affect the number of reference memory errors recorded over 14 days of testing (Treatment: F(3, 20) = 1.87; *p* > 0.16, NS). For all groups, the number of errors decreased significantly as training progressed (F13, 260) = 10.17; *p* < 0.0001), and the slope of the learning curve was similar (Treatment x Day interaction: F(39, 260) < 1, NS). The number of hits (Figure 6B), which can be taken as a relevant index of spatial discrimination performance, increased significantly from Day 1 to Day 14 (F13, 260) = 8.99, *p* < 0.001) and evolved similarly for all groups (Treatment x Day interaction: F(39, 260) < 1, NS). However, there was a main effect of EMF exposure (Treatment: F(3, 20) = 4.07, *p* < 0.03). Subsequent analyses revealed that, although the 1 W/kg exposed group did not differ from the control group (F < 1; NS), it significantly differed from the sham-exposed group (F(1,10) = 3.45; *p* < 0.05). This difference is likely due to the fact that the 1 W/kg group exhibited a lower number of hits than the sham group on Days 3 and 8 (*p* < 0.05). However, this difference between animals exposed to 1 W/kg and sham-exposed animals was not found on the entire population of animals used in this study (n = 12 per group; Supplementary Figure S2). When the whole population of animals was considered, there was neither a “treatment” effect, nor any interaction between the “day” and “treatment” factors (Fs < 1; NS).

As illustrated in Figure 6C, the latency to find the goal box was also similar across groups (Treatment: F(3, 20) = 1.15, *p* > 0.35, NS) and decreased in a similar fashion over days (Day: (F(13, 260) = 51.41, *p* < 0.0001); Treatment x Day interaction: F(39, 260) = 1.30; *p* > 0.12, NS). A more detailed analysis performed on the four trials of a daily session did not show any significant differences between groups, whatever the day of learning considered, thus indicating that learning that occurred within a session was similar across groups (data not shown). Overall, there was no evidence of an effect of EMF exposure in this task.

#### 3.2.2. c-Fos Mapping

##### Working Memory Task

**Figure 7:**
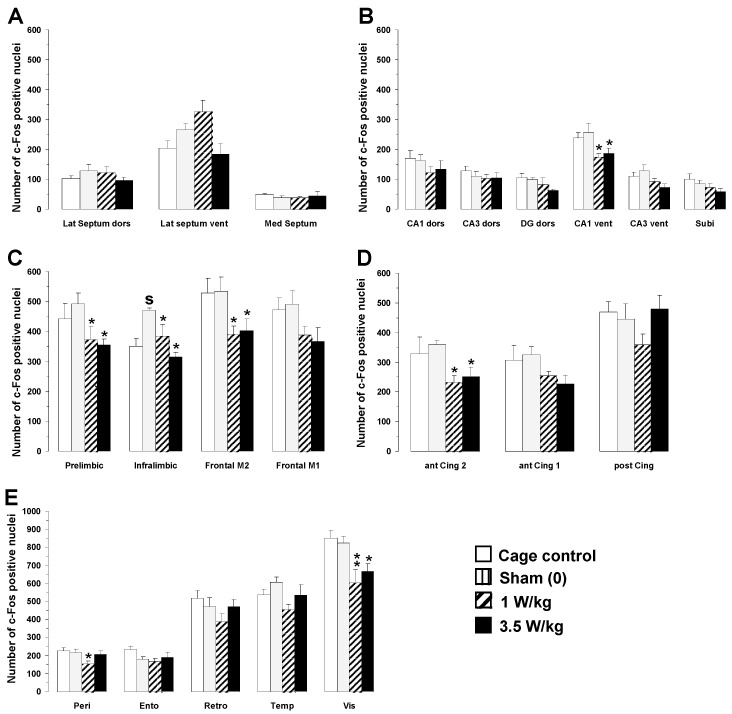
Effects of subchronic GSM 900 MHz electromagnetic field exposure on neuronal activity as measured by c-Fos immunohistochemistry in cage control, exposed, and sham-exposed animals submitted to the working memory task. Data are presented as number of c-Fos-positive nuclei in selected brain regions (mean ± SEM, n = 6/group). (**A**) Septal region; (**B**) hippocampal formation; (**C**–**E**) cortical regions. See Figure 2 for the complete list of abbreviations. Cage control rats were not submitted to any treatment before each daily training session. Sham-exposed rats were placed in the rockets for 45 min without any EMF exposure immediately prior to each testing session. Experimental animals were exposed to EMFs for 45 min at SARs of either 1 or 3.5 W/kg immediately prior to each testing session. * *p* < 0.05, ** *p* < 0.01 for exposed versus sham-exposed animals. ^S^ *p* < 0.05 for sham-exposed versus cage control animals, ANOVA followed by Fisher’s post hoc test.

As a general rule, no significant differences in c-Fos expression were observed between cage control animals and sham animals placed in the rockets without any EMF exposure. As shown in Figure 7, the only increase after sham exposure was observed in the infralimbic area of the prefrontal cortex (+33.9%; *p* < 0.01). A two-way ANOVA performed on c-Fos mapping data with BASARs as a between-group factor and the 23 brain regions analyzed as a within-subjects factor indicated a significant group x region interaction (F(3, 500) = 3.87; *p* < 0.01), showing that region-specific changes in c-Fos expression occurred following EMF exposure. Analysis of individual regions revealed that EMF exposure induced a decrease in c-Fos expression in the following brain regions: hippocampus (CA1 ventral part: (F(3, 20) = 3.56; *p* < 0.05), prefrontal (prelimbic area: F(3, 20) = 3.10; *p* < 0.05; infralimbic area: F(3, 20) = 6.97; *p* < 0.05), frontal (M2 motor area: F(3, 20) = 3.34; *p* < 0.05), anterior cingulate (area 2: F(3, 20) = 3.08; *p* < 0.05), perirhinal: (F(3, 20) = 3.12; *p* < 0.05), and visual cortices (F(3, 20) = 5.76; *p* < 0.01). 

**Figure 8:**
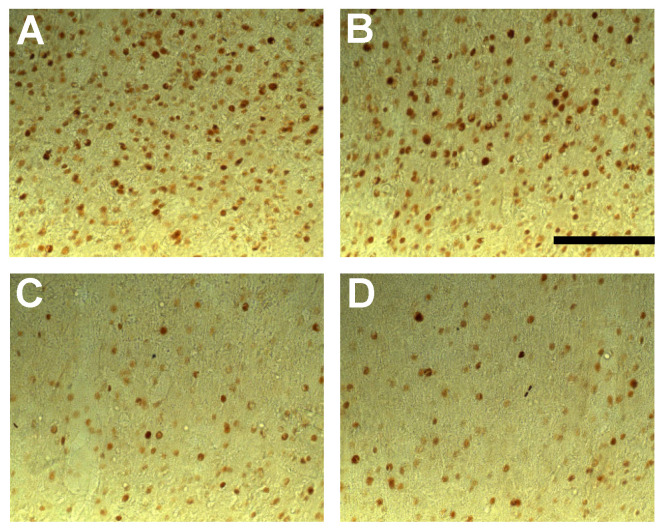
Photomicrographs (×20 magnification) of c-Fos immunoreactivity in coronal sections taken through the frontal cortex (M2 area) of animals submitted to the working memory task for 10 days and exposed daily to GSM 900 MHz electromagnetic fields (SAR of 1 or 3.5 W/kg). Exposure was given for 45 min immediately prior to each testing session. Note that this example represents the largest decreased number of c-Fos-positive nuclei in exposed animals (1 and 3.5 W/kg; (**C**,**D**), respectively) as compared to cage control (**A**) and sham-exposed animals (0 W/kg; (**B**)). No significant differences were observed between cage control and sham-exposed animals. Scale bar: 100 μm.

Post hoc analyses revealed that EMF exposure significantly decreased c-Fos expression as compared to sham animals in the following regions (Figure 6): hippocampus (CA1 ventral part, BASAR of 1 W/kg −32.1%, *p* < 0.05; SAR of 3.5 W/kg −26.9%, *p* < 0.05), prefrontal (prelimbic area: BASAR of 1 W/kg −24.4%, *p* < 0.05; BASAR of 3.5 W/kg −27.9%, *p* < 0.05; infralimbic area: BASAR of 1 W/kg −18.1%, *p* < 0.05; BASAR of 3.5 W/kg −32.9%, *p* < 0.001), frontal (M2 motor area: BASAR of 1 W/kg −26.9%, *p* < 0.05; BASAR of 3.5 W/kg −24.7%, *p* < 0.05), anterior cingulate (area 2 BASAR of 1 W/kg −35.3%, *p* < 0.05; BASAR of 3.5 W/kg −29.9%, *p* < 0.05), perirhinal (BASAR of 1 W/kg −29.3%, *p* < 0.05), and visual cortices (BASAR of 1 W/kg −26.8%, *p* < 0.01; BASAR of 3.5 W/kg −19.1%, *p* < 0.05). Examples of the largest EMF-induced decreases in c-Fos expression in the frontal cortex are presented in Figure 8. 

##### Reference Memory Task

**Figure 9:**
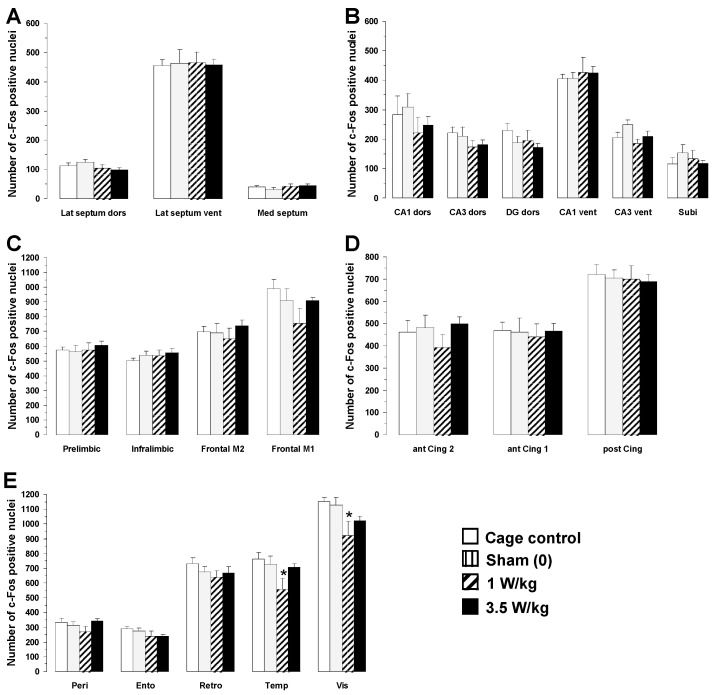
Effects of subchronic GSM 900 MHz electromagnetic field exposure on neuronal activity as measured by c-Fos immunohistochemistry in cage control, exposed, and sham-exposed animals submitted to the reference memory task. Data are presented as number of c-Fos-positive nuclei in selected brain regions (mean ± SEM, n = 6/group). (**A**) Septal region; (**B**) hippocampal formation; (**C**–**E**) cortical regions. See Figure 2 for the complete list of abbreviations. Cage control rats were not submitted to any treatment before each daily training session. Sham-exposed rats were placed in the rockets for 45 min without any EMF exposure immediately prior to each testing session. Experimental animals were exposed to EMFs for 45 min at SARs of either 1 or 3.5 W/kg immediately prior to each testing session. * p < 0.05 for exposed versus sham-exposed animals, ANOVA followed by Fisher’s post hoc test.

As shown in Figure 9, no significant differences in c-Fos expression were observed between cage control animals and sham animals placed in the rockets without exposure to EMFs. As for animals engaged in the working memory task, one-way ANOVA revealed that EMF exposure induced a dose-dependent decrease in c-Fos expression, but in only two brain regions: the temporal (F(3, 20) = 3.05; *p* < 0.05) and visual cortices (F(3, 20) = 3.30; *p* < 0.05). Post hoc analyses revealed that exposure to a BASAR of 1 W/kg significantly decreased c-Fos expression in the temporal cortex by 23.6% (*p* < 0.05) and in the visual cortex by 18.5% (*p* < 0.05) as compared to sham animals (Figure 9E).

### 3.3. Relations between Dosimetric Analysis and Changes in c-Fos Expression

In the two experiments presented here (i.e., both after acute and subchronic exposures), some brain areas exhibited changes in c-Fos labeling, whereas many others did not. Two possibilities could explain why only a few areas show changes in c-Fos labeling. First, these areas might be those where the local SAR values are the highest. Second, these areas might be those indirectly activated by EMF exposures via mechanisms that remain to be determined. 

**Figure 10:**
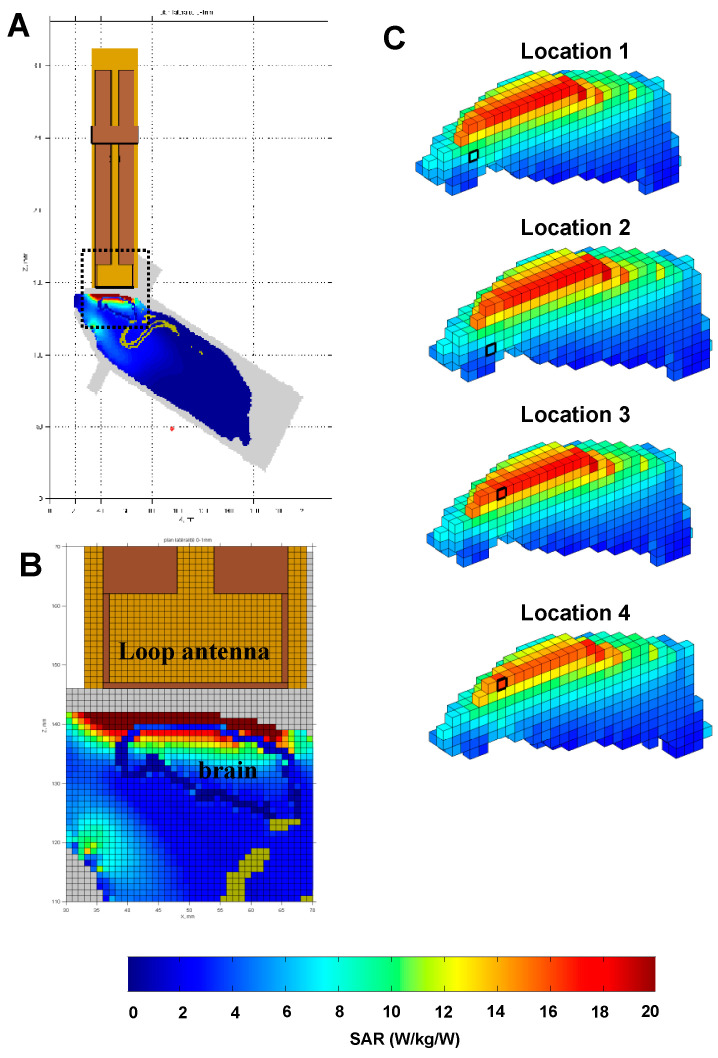
Dosimetric analysis of specific absorption rates (SARs) in the rat brain during exposure to GSM 900 MHz. The heterogeneous model of rat phantom and loop antenna described by Lévêque et al. [51] was used to evaluate the local SAR in the brain with a 1 mm cubic mesh. As explained by Lévêque et al. [51], an MRI of an adult rat (250 g) maintained in the rocket was carried out, and the tissues (skin, skull, brain, marrow, fat, muscles) were segmented and assigned to different dielectric properties. (**A**) Global view of the rat phantom in the rocket with the loop antenna above its head. The area defined by the dotted line is shown in (**B**). (**B**) This view shows the interface between the loop antenna and the rat’s head. It clearly indicates that the first millimeters of tissue below the loop antenna (i.e., the skin, the skull, the dura mater, and the cortex) received the highest SAR values. (**C**) Based on the relative location of the brain areas in the Paxinos and Watson atlas [55] and on their corresponding locations in the model of the rat brain, values of SAR for the brain areas exhibiting changes in c-Fos labeling were evaluated. The locations 1, 2, 3, and 4 depicted here correspond to the position of the prelimbic, infralimbic, frontal M2, and frontal M1 areas, respectively. Note that the two frontal areas (M1 and M2) received high SAR values, whereas the prelimbic and infralimbic areas received low SAR values because they are located deeper in the brain. Despite this important difference in terms of SAR values, they all expressed changes in c-Fos labeling.

To search for possible relationships between the SAR values and the cortical areas exhibiting changes in c-Fos activation after EMF exposure, we referred to SARs levels calculated in a non-homogeneous rat model with a 1 mm3 spatial resolution (detailed in [51]). Figure 10A,B provide global views of the rat brain model (with the loop antenna above) and show that the rat’s head absorbs 86% of the total power absorbed by the animal. The whole-body SAR was estimated to be 0.13 ± 0.34 W/kg. 

For eleven brain areas showing changes in c-Fos activation in our experiments (see Supplementary Table S1), the Paxinos and Watson [55] coordinates were converted in coordinates used for the rat brain model, and their local SARs were determined. Figure 10C presents the SAR distribution in the rat brain from four sagittal sections and indicates the location of four areas (black squares). It appears that the prelimbic and infralimbic areas (locations 1 and 2) received low SAR values, whereas the two frontal areas M2 and M1 (locations 3 and 4) received high SAR values. Yet, these four areas exhibited changes in c-Fos labeling during resting conditions (Experiment 1) and during behaviorally induced c-Fos activation (Experiment 2). Thus, there was no evidence for systematic relationships between the SAR level in the rat brain and the changes in c-Fos labeling after EMF exposure.

## 4. Discussion

The aim of the present study was to evaluate, in a rat model, the potential consequences (positive or negative) of exposure to 900 MHz GSM signals. To mimic as closely as possible the use of cellular phones, we used a head-only exposure system allowing application of calibrated intensities of EMFs at the vicinity of the animal’s head. Mapping of brain regions affected by EMF exposure was achieved by measuring the expression of the activity-dependent gene c-Fos, which is rapidly induced after external stimulation. As c-Fos expression and behavioral performance can both be affected by stressful conditions [56,57], special care was taken to minimize as much as possible the stress effect induced by the confinement in exposure rockets. In the two experiments, we progressively habituated the animals to be restrained to reduce stress responses. This procedure was probably adequate because in Experiment 2, no differences in c-Fos expression (in all but one brain area) and in behavioral performance were found between cage control animals (maintained in their home cage) and sham animals (placed in the rockets but not exposed to EMFs). Although cage control animals were not used in Experiment 1, the extended habituation (two weeks vs. one week in Experiment 2) should have largely reduced potential effects resulting from the restrained conditions. 

In the first experiment, a 2 h acute exposure to 900 MHz EMF differentially affected the brain regions analyzed in animals at rest. Out of the 23 brain areas investigated, only six showed significant changes in c-Fos labeling with a dose-dependent increase for five of them at a BASAR of 1 W/kg. In the second experiment, after 10 or 14 days of 45 min exposure to 900 MHz EMF delivered immediately before behavioral testing, six brain areas displayed a significant decrease in c-Fos labeling. It is quite striking that four of these brain areas were cortical areas exhibiting increases in c-Fos labeling after a 2 h acute exposure. Surprisingly, our dosimetric analysis indicated that among the brain areas showing changes in c-Fos labeling, some areas received high SAR values, whereas others corresponded to low SAR values. 

### 4.1. Relation with Previous Studies Evaluating the Effects of Acute Exposure to EMFs

Only a few studies have addressed the possibility that mobile phone radiations could induce genomic responses in vivo. Using head-only exposures, a slight increased expression of c Fos messenger RNA was observed by Fritze and colleagues [28] in some brain areas (cerebellum, neocortex, and piriform cortex) after 4 h of GSM 900MHz exposures to various BASARs (0.3, 1.5, and 7.5 W/kg). As these increases were also detected on sham-exposed animals, they most likely result from the restrained conditions used during EMF exposure. Furthermore, hyperthermia that has been measured in most rats following whole-body exposure to SAR > 5 W/kg could explain the prominent c-Fos protein expression observed in several cortical and periventricular areas [58]. Different upstream regulatory elements could mediate an increase in c-Fos expression: changes in neurotransmitters releases, increase in Ca++ influx, or neurotrophic factors. Also, emerging evidence suggests that c-Fos is essential in regulating neuronal cell survival versus death [59]. Previous in vitro studies have reported that different microwave exposures (continuous or pulsed) influence signal transduction pathways in brain tissue [60,61]. More recent studies have also reported changes in gene expression after various types of EMF exposures [62,63,64,65,66]. However, the cellular mechanisms triggered by GSM exposure such as transcriptional activity, activation of specific signaling pathways, or immune responses can differ between rats and humans. But obviously, any potential effects observed in animals’ studies should be used to guide the research in human subjects. Lastly, these experimental animal models often used EMFs quite different from the GSM signals and, therefore, the results should be interpreted with caution. 

As the c-Fos immediate early gene is not considered a marker of cerebral injury, changes in c-Fos expression observed following EMF exposure do not necessarily indicate a deleterious effect of EMFs on brain metabolism. Of note, however, and contrary to what was initially believed, glial cells (including microglia, astrocytes, and oligodendrocytes) have been found to express Fos proteins [67]. In astrocytes, c-Fos expression can thus be the consequence of inflammatory processes via the recruitment of the ERK and/or p38MAPK pathway and the subsequent phosphorylation of Elk1 at the c-Fos promoter, raising the possibility that exposure to 900 MHz GSM signals may be perceived by the cerebral tissue as a potentially harmful stimulus. Further investigation is needed, however, to unravel in which cell types Fos expression occurs following EMF exposure. Other mechanisms are also likely to contribute to increase Fos expression following EMF exposure. For instance, cerebral changes in heat shock protein (HSP) expression were reported in rats exposed to GSM-900 MHz and GSM-1800 MHz signals [68], raising the possibility that GSM-induced HSP signaling may upregulate Fos expression and contribute to the c-Fos increases observed in Experiment 1. Functionally, these increases may underlie an adaptive process of the brain tissue to stress. Nonetheless, it is important to mention at recent in vitro electrophysiological studies [69,70] found decreases in spontaneous activity when exposing neuronal cultures to 1800 MHz or 3.5 GHz signals. In addition, no alteration of spontaneous and evoked activity was detected in vivo when healthy rats were exposed for 2 h to 1800 MHz, whereas decreases were detected in under acute neuroinflammation induced by lipopolysaccharide [71,72].

In a few in vivo studies using a head-only exposure system similar to ours, molecular and cellular alterations of brain cells have been described after acute exposures to 900 MHz GMS signals. First, a 2 h exposure at BASARs of 4 and 32 W/kg decreased the content in gamma-aminobutyric acid (GABA) in the cerebellum [24]. Second, a significant reduction in the number of NMDA receptors at the post-synaptic level has been observed in cortex and striatum after a brief (15 min) exposure to 900 MHz GSM signals [27]. Also, increases in glial immunoreactivity have been reported in the cortex, striatum, and cerebellum [26,27]. However, these latter effects were obtained at quite high BASAR levels (32 W/kg and 6 W/kg), out of the range of normal SAR delivered by cell phones. Some of these effects, particularly those on glial reactivity, were not replicated at lower BASAR values of 2–3 W/kg. Indeed, in previous studies performed both in developing or in adult rats, we did not observe tissue damage nor microglial or astroglial activation after a 2 h of exposure to GSM in healthy animals [29,66,71,72]. In our experimental conditions, we could not detect increases in local brain temperature (in area FR2) at a SAR of 1 W/kg and only modest increases (less than 0.5 °C) at a SAR of 3.5 W/kg (Dubreuil, Jay, and Edeline, unpublished results).

### 4.2. Relationship with Previous Studies Evaluating the Effects of Subchronic Exposure to EMFs

Though interesting, the effects observed following “acute” EMF exposure are not the most relevant because EMF exposure through cell phones is almost universally chronic (over years) in the human population. In fact, the immediate effects of acute EMF exposure could dissipate quickly through compensatory mechanisms during long-term exposure, and repeated exposure could itself induce homeostasis effects. Here, after daily 45 min exposures to EMFs, behavioral performance was not impaired in a working memory task, and the minor impairment observed in the reference memory task on a subset of animals was not confirmed on a larger size population (n = 12 animals/group). These data replicate our original observations [43], which have been corroborated by other laboratories [39,40,42,45]. Exposing animals to behavioral tasks had two main advantages. First, it allowed for the detection of possible decreases in c-Fos expression, which would be otherwise difficult to observe in resting animals (because of floor effect). Second, it allowed for the detection of changes in the capacity of certain brain regions to be activated in response to cognitive demand. Regarding the first advantage, we observed that the level of c-Fos expression in the sham-exposed rats differed in the acute versus the subchronic exposure experiment. For example, the mean value obtained for the prelimbic area was about 310 c-Fos-positive nuclei/mm^2^ in Experiment 1 (acute exposure), whereas it was 495 and 560 (in the working memory and in the reference memory task, respectively) in Experiment 2 (subchronic exposure). These large increases (60% and 80%) in c-Fos labeling in sham animals probably indicate the involvement of several brain areas in the behavioral tasks during Experiment 2. 

Two hypotheses could be proposed to account for the decrease in c-Fos labeling observed in exposed animals during the second experiment. First, one could consider that neurons, as any biological tissue [73,74], adapted to repeated exposure to EMF, as well as responding differently to an acute vs. a subchronic exposure. Thus, although the behavioral tasks increased c-Fos expression in all animals, this increase was attenuated in the animals exposed daily to the EMF because the repetition of exposures produced opposite effects when compared with a single exposure. However, according to this hypothesis, we should have obtained the same decreases after the two behavioral tasks, which was not the case. A second explanation relies on the fact that performing a behavioral task produced a “cognitive demand” triggering the activation of several cortical networks, an activation which was attenuated by prior exposure to the GSM EMFs. This possibility may explain why the results differed between the two behavioral tasks: In the easiest task, the reference memory task, EMF-exposure-induced decreases were limited to temporal and visual cortices. In contrast, in the working memory task, animals exposed to EMFs exhibited a reduction in the number of c-Fos-positive nuclei in many cortical regions (prelimbic, infralimbic, frontal, cingulated, perirhinal, and visual cortices; area CA1 in hippocampus), suggesting the recruitment of a larger network of brain areas during this task.

As the c-Fos protein is widely considered as an indirect correlate of neuronal activation [67,75], the present data point out that daily exposure to GSM signals attenuates the increase in neuronal activity normally observed in brain areas during a behavioral task. However, this hypo-activity was not sufficient to induce detectable concomitant deficit performance in the working and reference memory tasks used here. One may argue that the use of more challenging behavioral tests might have revealed cognitive impairment following EMF exposure. However, we could not find impairments either by using a more complex working memory task (introducing a 10 s confinement between the visit of each arm or a 15 min delay after four visited arms) or by increasing the temporal delay between the acquisition and the testing period in an object recognition task [42]. Several additional experiments would be necessary to totally discard the possibility that the decrease in c-Fos labeling detected here does not lead to behavioral deficits. For example, the use of a longer retention interval associated with a reference memory task (e.g., retention sessions given several weeks after the end of acquisition) could reveal detectable retrieval deficits. It is also possible that longer subchronic exposures (over weeks) might reveal behavioral impairments with the same paradigms used here (see, for example, the results obtained by [76,77,78,79]). However, we should also keep in mind that in some cases, positive effects of EMF exposure have been detected after chronic EMF exposure both in normal and pathological conditions [48,49].

Cellular alterations of brain cells have been reported after subchronic exposures to 900 MHz GSM signals. A decrease in cytochrome oxydase activity was detected in several cortical areas (including the frontal areas) after a 7 day exposure to BASARs of 6 W/kg but not after a similar duration of exposure to BASARs of 1.5 W/kg [80,81]. Longer exposures (5 days a week for 24 weeks) also induced increases in GFAP immunostaining in several brain areas (frontal cortex, dentate gyrus, striatum) at a BASAR of 6 W/kg but not at a BASAR of 1.5 W/kg [25,26]. Using low BASAR levels (1.5 and 3.5 W/kg), the present data show that exposure to EMFs does have a biological effect on neurons but also indicates that changes in neuronal activity (as revealed by c-Fos imaging) do not necessarily lead to behavioral impairments. As cytochrome oxidase activity is an endogenous metabolic marker, the effects that it can reveal (as well as those revealed by GFAP immunostaining) might reflect more permanent changes obtained when the neural tissue is chronically exposed to higher SARs (6 W/kg in the case of the studies by Ammari and colleagues [25,80,81]). The main objective of our study was to track activity-dependent changes induced by 900 MHz GSM exposures. We however acknowledge limitations related to the use of our approach. For instance, we have not performed double-staining and cannot determine whether c-Fos is differentially expressed across cell types (excitatory or inhibitory neurons, glial or endothelial cells) following EMF exposure. A dedicated transcriptomic analysis using single-cell RNA sequencing from different components of the brain tissue would be worth considering in future studies. Also, our study was restricted to male rats due to the impossibility of separately housing male and female rats during GSM 900 MHz exposure and behavioral testing. Whether female rats could be more sensitive to GSM 900 MHz exposure would deserve further investigation, although data from animal and human studies have not pinpointed an apparent gender effect.

## 5. Conclusions and Open Questions

Several questions remain open after the present study. The first and more puzzling one is the dose-dependent increase in c-Fos labeling observed in Experiment 1: in five out of six brain areas, the BASAR level that produces an increase in c-Fos labeling was not the highest BASAR tested (6 W/kg) but the BASAR of 1 W/kg. The existence of windows for the effects of EMFs (either for the frequency, the duration, or the intensity of the EMF) has long been debated, and the cellular bases of these effects are still unclear (see for review [82,83,84]. In the range of SAR used here, it was shown that the incidence of DMBA-induced mammary gland tumors was increased by subchronic exposure to 900 MHz GSM at a SAR of 1.4 W/kg but not at 2.2 or 3.5 W/kg [85]. In vitro studies indicate (i) that neurons are at least 10 times more sensitive than previously thought to weak electric fields [86,87]; (ii) that electrophysiological effects on neuronal activity can be obtained at very low SARs (0.0016–0.0044 W/kg); and (iii) that within this range, the effects can differ as a function of the field intensity [88]. The second unresolved question is why some brain areas expressed changes in c-Fos labeling whereas others did not. The dosimetric analysis allows for the discarding of the possibility that changes in c-Fos labeling only occur in brain areas receiving the highest SARs. However, it is possible that unspecific stimuli, such as local thermal stimuli occurring during exposure within the skin or the dura, activated brain regions even if these brain regions did not receive the highest SARs. This possibility should be kept in mind because many studies in humans have shown that noxious or innocuous thermal stimuli activate cortical areas such as the anterior cingulate cortex and other regions of the frontal cortex [89,90,91,92] that are similar to those showing changes in c-Fos labeling in our experiments. Last, it is also possible that some brain areas are more sensitive to EMF than others. In this context, it is important to mention that, in humans, a 30 min exposure to 900 MHz GSM signals (BASAR 1 W/kg) has been shown to increase the relative regional cerebral blood flow in the dorsolateral prefrontal cortex on the side of exposure [93]. Even the low-field magnetic stimulation (920 Hz) used in echo planar imaging for functional MRI studies seems capable of modifying brain glucose metabolism in localized brain regions including frontal and parietal cortices [94]. By showing that significant effects can be obtained at low SAR levels, the present results, as well as other animals studies reviewed in [95,96], obviously call for more systematic investigations looking for changes in neuronal activity after long-duration exposures to low SAR levels.

## Data Availability

The data presented in this study are available upon request from the corresponding author.

## Supplementary Materials

The following supporting information can be downloaded at https://www.mdpi.com/article/10.3390/biomedicines12091954/s1.
