# Supplementary material for: Effects of Head-Only Exposure to 900 MHz GSM Electromagnetic Fields in Rats: Changes in Neuronal Activity as Revealed by c-Fos Imaging without Concomitant Cognitive Impairments
Source: Biomedicines. 2024 Aug 27;12(9):1954. doi: 10.3390/biomedicines12091954 (PMC11428239; doi:10.3390/biomedicines12091954)
Supplement: Supplementary file 1 [file biomedicines-12-01954-s001.zip › biomedicines-3137719-supplementary.pdf]

A.

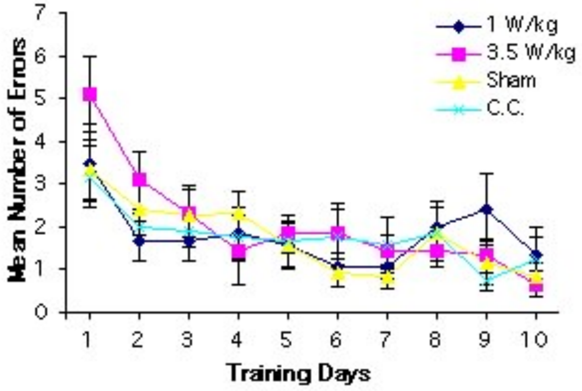

B.

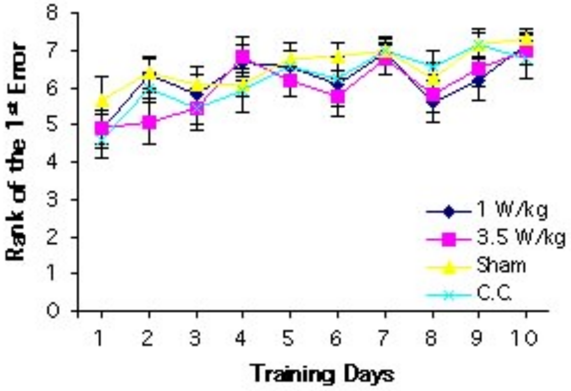

Supplementary Figure S1:
Performance in radial-arm maze (working memory task) for the total population of animals (n=12/group):
(A) The mean number of errors decreased from day to day for all the treatment groups. There was no significant difference between the groups.
(B) The rank of the first error increased from day to day for all groups.
The learning curves of exposed and non-exposed rats are similar. As assessed by the number of errors and the rank of the first error, all groups learned in a similar way.

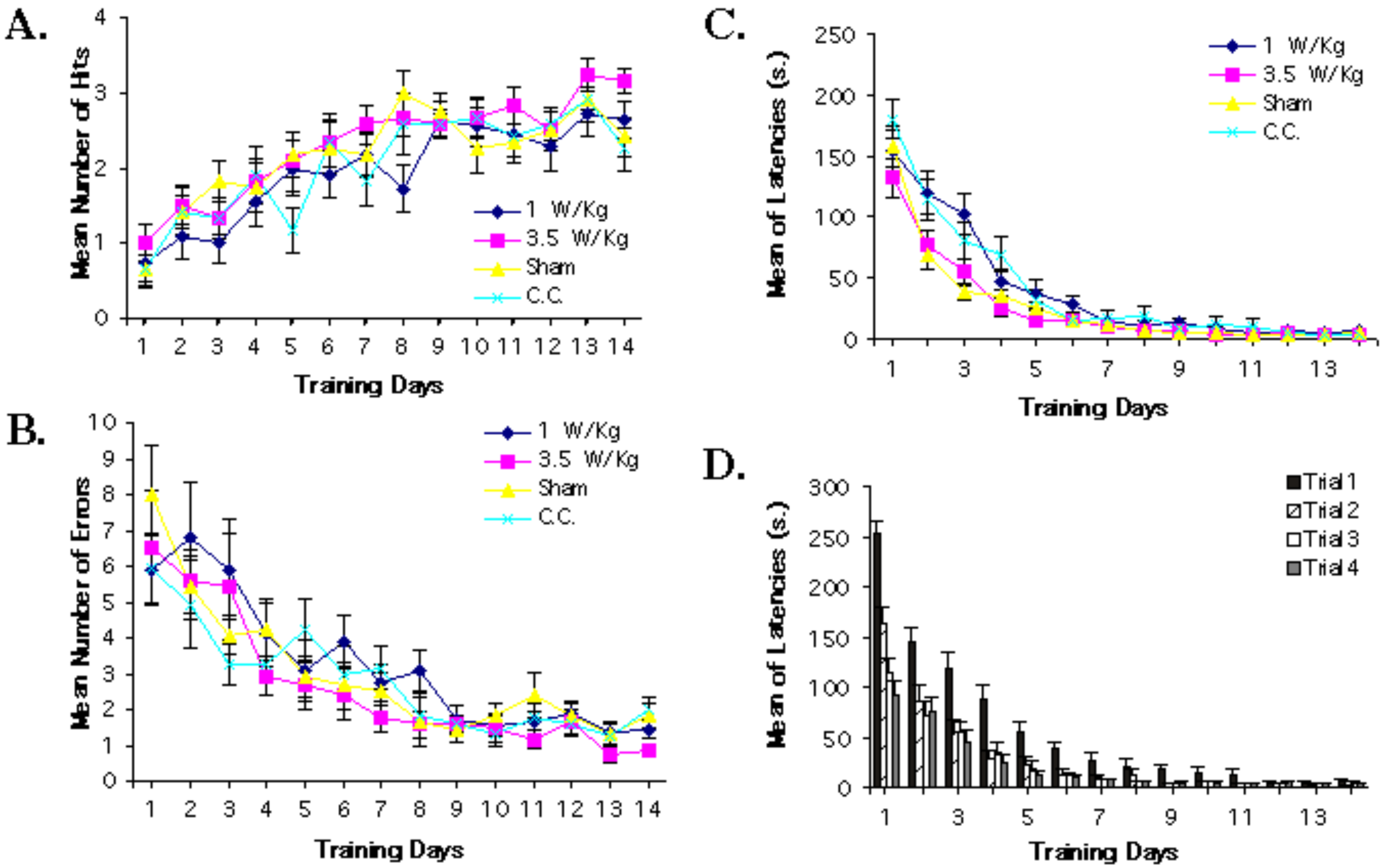

Supplementary Figure S2: Performance of the whole population of rats (n=12/group in the reference memoy task.
(A) The number of hits increased over the whole training period for all the groups. There was no significant difference between the learning performance of the treatment groups. (B) The number of errors decreased during the training period for all groups. There was no significant difference between the learning performances of the treatment groups. (C) The latencies decreased similarly for all treatment groups. (D) The latency of the first trial was always longer than the other trial latencies (warm-up decrement effect). The difference between the latency of the first trial and the other latencies diminished over the days and this attenuation was similar for the treatment groups.

Supplementary Table S1

	Anteriority	Laterality	Depth	SAR W/kg/W	
PrL Area	4-5 mm	0-1 mm	3-4 mm	7.80	Point 1
IL Area	4-5 mm	0-1 mm	4-5 mm	5.74	Point 2
FM2 Area	5,7-6.7 mm	0-1 mm	0-1 mm	16.44	Point 3
FM1 Area	5.7-6.7 mm	2-3 mm	0-1 mm	16.06	Point 4
Dorsal Septum	5.7-6.7 mm	0-1 mm	3.5-4.5 mm	8.90	Point 5
Ventral Septum	5.7-6.7 mm	0-1 mm	5-6 mm	5.75	Point 6
Cg2 Area	6.7-7.7 mm	0-1 mm	2-3 mm	10.58	Point 7
Parietal Area	9.5-10.5 mm	5-6 mm	0-1 mm*	8.72	Point 8
Subiculum	12-13 mm	1-3 mm	3-4 mm	8.37	Point 9
Visual Area	12-13 mm	5-6 mm	0-1 mm*	7.72	Point 10
Temporal Area	12-13 mm	6.5-7.5 mm	0-1 mm*	7.720	Point 11
